# Nuclear-encoded factors associated with the chloroplast transcription machinery of higher plants

**DOI:** 10.3389/fpls.2014.00316

**Published:** 2014-07-03

**Authors:** Qing-Bo Yu, Chao Huang, Zhong-Nan Yang

**Affiliations:** ^1^Department of Biology, College of Life and Environmental Sciences, Shanghai Normal UniversityShanghai, China; ^2^Institute for Plant Gene Function, Department of Biology, Shanghai Normal UniversityShanghai, China

**Keywords:** plastid transcription, RNA polymerase, PEP, NEP, functional modules

## Abstract

Plastid transcription is crucial for plant growth and development. There exist two types of RNA polymerases in plastids: a nuclear-encoded RNA polymerase (NEP) and plastid-encoded RNA polymerase (PEP). PEP is the major RNA polymerase activity in chloroplast. Its core subunits are encoded by the plastid genome, and these are embedded into a larger complex of nuclear-encoded subunits. Biochemical and genetics analysis identified at least 12 proteins are tightly associated with the core subunit, while about 34 further proteins are associated more loosely generating larger complexes such as the transcriptionally active chromosome (TAC) or a part of the nucleoid. Domain analyses and functional investigations suggested that these nuclear-encoded factors may form several functional modules that mediate regulation of plastid gene expression by light, redox, phosphorylation, and heat stress. Genetic analyses also identified that some nuclear-encoded proteins in the chloroplast that are important for plastid gene expression, although a physical association with the transcriptional machinery is not observed. This covers several PPR proteins including CLB19, PDM1/SEL1, OTP70, and YS1 which are involved in the processing of transcripts for PEP core subunit as well as AtECB2, Prin2, SVR4-Like, and NARA5 that are also important for plastid gene expression, although their functions are unclear.

## Introduction

Plastids are specific organelles in plant and algal cells that are responsible for photosynthesis and some important metabolic pathways. They possess their own genetic material and are generally considered to be of endosymbiotic origin (McFadden and van Dooren, 2004). Similar to bacteria, the DNA is organized into dense particles, the nucleoids (Pfalz and Pfannschmidt, 2013). The genome size from plastids of vascular plants ranges from 120 to 180 kbp and the encoded gene set is highly conserved (Sugiura, 1992). They can be categorized into three groups according to their molecular function of the encoded components: (1) Components of the plastid gene expression machinery (RNA polymerase, ribosomal proteins, tRNAs, and rRNAs); (2) Subunits of photosynthesis-related complexes (Rubisco, PSII, the cytochrome b6f complex, PSI, NAPH dehydrogenase, and ATP synthase), and (3) a few proteins involved in other processes (e.g., ClpP1 and YCF3) (Sugiura, 1992). The chloroplast proteome is estimated to be between 2100 and 3600 proteins (Leister, 2003). Most of the chloroplast proteins are encoded by the nuclear genome and are imported from the cytosol (Li and Chiu, 2010), due to the limited coding capacity of the chloroplast genome. However, chloroplast gene expression is still essential for the development of chloroplasts and the maintenance of chloroplast functions. It involves the action of numerous nuclear-encoded factors, besides proteins encoded by the plastome. Recently, proteomics data (Pfannschmidt et al., 2000; Ogrzewalla et al., 2002; Suzuki et al., 2004; Pfalz et al., 2006; Steiner et al., 2011; Melonek et al., 2012) and genetic analysis (Chi et al., 2008; Ogawa et al., 2009; Wu and Zhang, 2010; Qiao et al., 2011, 2013; Kindgren et al., 2012; Pyo et al., 2013; Yu et al., 2013) identified that numerous nuclear-encoded proteins with various functions are associated with the transcriptional machinery and are involved in chloroplast gene expression. In this paper, we focused on these nuclear-encoded factors for chloroplast transcription.

### Two types of plastid RNA polymerases in higher plants

Plastid genes are transcribed by two RNA polymerases, the nuclear-encoded RNA polymerase (NEP) and the plastid-encoded RNA polymerase (PEP). NEP is a phage-type RNA polymerase with a single subunit (Chang et al., 1999; Lerbs-Mache, 2011). In *Arabidopsis*, the nuclear genome encodes three NEPs. RpoTp is targeted to chloroplast, RpoTm is targeted to mitochondria, and RpoTmp is dually targeted to both organelles (Hess and Borner, 1999). NEP is important for plant development. Inactivation of RpoTp results in defects in plastid gene expression and leaf development (Hricová et al., 2006; Swiatecka-Hagenbruch et al., 2008) while plants with inactivated RpoTmp exhibit several defects, including a plastid gene expression defect, delayed greening and growth retardation of leaves and roots (Courtois et al., 2007). The dysfunction of both NEPs resulted in seedling lethality at a very early developmental stage (Hricová et al., 2006). Although NEP is generally considered to be a single subunit RNA polymerase, recent biochemical analysis revealed that RPOTmp interacts with a thylakoid RING-H2 protein. This protein might mediate the fixation of RPOTmp to thylakoid membranes in order to regulate the transcription of the plastid *rrn* genes (Azevedo et al., 2008).

PEP is composed of four core subunits encoded by the genes *rpoA, rpoB*, *rpoC1*, and *rpoC2* that are located on the plastid genome. PEP exhibits a certain sensitivity to inhibitors of bacterial transcription, such as tagetitoxin, and the group of rifampicin-related drugs, indicating a distinct degree of conservation of these eubacterial-type RNA polymerase during evolution (Liere et al., 2011). Like for bacterial RNA polymerases, the activity/specificity of the PEP core enzyme is regulated by sigma-like transcription factors that are encoded by the nuclear genome of higher plants. In *Arabidopsis*, there exist six chloroplast sigma factors (SIG1–SIG6). These sigma factors might have overlapping as well as specific functions for recognizing a specific set of promoters during chloroplast development (Schweer, 2010; Liere et al., 2011). Besides the sigma factors, however, the core subunits of PEP are associated also with additional proteins (see below) that mediate a number of additional functions to the PEP complex.

NEP and PEP play different roles in plastid gene transcription during plastid development and plant growth (Liere et al., 2011). Based on their transcription by the different RNA polymerases, plastid genes can be grouped into three classes (Hajdukiewicz et al., 1997; Ishizaki et al., 2005). Transcription of photosynthesis-related genes (such as *psbA*, *psbD*, and *rbcL*) depend largely on PEP (class I), whereas a few house-keeping genes (mostly encoding components of the transcription/translation apparatus, such as *rpoB*) are exclusively transcribed by NEPs (class III). Most of plastid genes, however, are transcribed by both PEP and NEPs (class II). Generally, NEP is more active in the young, non-green tissues early in leaf development. It transcribes housekeeping genes including the four core subunits of PEP polymerase which primarily constitute the plastid gene expression machinery. Once PEP is formed in later developmental stages, it thereafter transcribes the photosynthesis-related genes (Hajdukiewicz et al., 1997; Lopez-Juez and Pyke, 2005; Schweer et al., 2010b) and plastid tRNAs (Williams-Carrier et al., 2014). In the mature chloroplast, the activity of NEP is barely detected, while PEP activity maintains high for chloroplast development and plant growth. Nevertheless, recent investigations demonstrated that both NEP and PEP are present in seeds, and PEP is also important for seed germination. This indicates that PEP exists also in non-photosynthetically active seed plastids (Demarsy et al., 2006).

### PEP is associated with numerous nuclear-encoded proteins

Early biochemical analysis demonstrated that two different forms of the PEP complex exist in higher plant, that is, PEP-A and PEP-B (Pfannschmidt and Link, 1994). PEP-B is composed only of the *rpo* core subunits and is present in both etioplasts and greening chloroplasts. During light-dependent chloroplast development, this PEP-B enzyme is reconfigured into an eukaryote-like enzyme complex, the PEP-A, by association of numerous proteins (Pfannschmidt and Link, 1997; Steiner et al., 2011; Pfalz and Pfannschmidt, 2013). PEP-A is the major RNA polymerase in matured chloroplast of higher plant. Attempts have been focused on the isolation of the plastid RNA polymerase complex and its associated proteins for many years (Pfalz and Pfannschmidt, 2013). Biochemical analyses uncovered that the core *rpo* subunits of PEP are present in both the insoluble RNA polymerase preparation called transcriptionally active chromosome (TAC), and the soluble RNA polymerase preparation (sRNAP) (Krause and Krupinska, 2000; Pfalz et al., 2006; Melonek et al., 2012). The TAC fraction was isolated from lysed plastids through one or two gel filtration chromatography steps and subsequent ultracentrifugation, while the soluble RNA polymerase (sRNAP) is prepared from isolated and lysed plastids *via* several chromatographic purification steps without precipitation by centrifugation (Pfalz and Pfannschmidt, 2013). Based on gel filtration and mass spectrometry analysis from different organisms, including *Nicotiana tabacum* (Suzuki et al., 2004), *spinach* (Melonek et al., 2012), *mustard* (*Sinapis alba*) (Pfannschmidt et al., 2000; Pfalz et al., 2006; Steiner et al., 2011), and *Arabidopsis* (Pfalz et al., 2006) it is estimated that the TAC complex contains 43 nuclear-encoded proteins (Table 1). Ten proteins were reproducibly found to be tightly associated with PEP core subunits in *mustard* seedlings and, therefore, were named polymerase-associated proteins (PAPs) (Steiner et al., 2011). The other proteins were found in the previous reported TAC complex and might represent more loosely attached components of the transcription machinery (Pfalz et al., 2006). Two TAC components, pTAC7 (Yu et al., 2013) and MurE-like (Garcia et al., 2008), were not identified as PAPs in *mustard* (Steiner et al., 2011), however, based on their mutant phenotype in T-DNA inactivation mutants of *Arabidopsis* these two proteins were proposed to be PAPs (Pfalz and Pfannschmidt, 2013). One essential common feature of all PAPs is that they are essential for PEP activity. The *Arabidopsis* knock-out lines for the corresponding genes show all an albino/ivory or pale-green phenotype with severe defects in chloroplast development and PEP-dependent transcription (Table 1) (Pfalz et al., 2006; Garcia et al., 2008; Myouga et al., 2008; Arsova et al., 2010; Schröter et al., 2010; Gao et al., 2011; Steiner et al., 2011; Gilkerson et al., 2012; Yagi et al., 2012; Yu et al., 2013). The phenotype of these PAP mutants is identical to that of *rpo*-gene knock-out mutants in tobacco (Allison et al., 1996; Hajdukiewicz et al., 1997; De Santis-MacIossek et al., 1999). In the knockout mutants of AtECB1/SVR4/MRL7 (Qiao et al., 2011; Yu et al., 2014), PEP-Related Development Arrested 1 (PRDA1) (Qiao et al., 2013), and Delayed Greening 1 (DG1) (Chi et al., 2008), the expression of PEP-dependent chloroplast genes is also severely reduced. These proteins have not been identified in PEP complex by previous proteomic analyses (Krause and Krupinska, 2000; Suzuki et al., 2004; Pfalz et al., 2006; Steiner et al., 2011). Nevertheless, they interacts with some members of the PEP/TAC complex (Chi et al., 2010; Qiao et al., 2011, 2013; Kindgren et al., 2012; Yu et al., 2014) and are either loosly or temporarily attached.

**Table 1:** **Proteomics Identification of Chloroplast PEP complex Components in Higher Plant**.

**Gene name**	**AGI number**	Species^*^	Localization^**^	Molecular phenotype^*****^	Protein domain^***^	Molecular function^****^	**References**
PAP1/pTAC3^a^	AT3G04260	*Arabidopsis*/*Mustard*	Chloroplast nucleoid^b^	Low PEP activity	SAP domain	DNA binding ability	Pfalz et al., 2006; Steiner et al., 2011; Yagi et al., 2012
PAP2/pTAC2^a^	AT1G74850	*Arabidopsis*/*Mustard*	Chloroplast nucleoid^b^	Low PEP activity	pentatricopeptide repeat protein	Unknown	Pfalz et al., 2006; Steiner et al., 2011
PAP3/pTAC10^a^	AT3G48500	*Arabidopsis*/*Mustard*	Chloroplast nucleoid^b^	Low PEP activity	S1 domain	RNA binding ability	Pfalz et al., 2006; Steiner et al., 2011; Jeon et al., 2012
PAP4/FSD3^a^	AT5G23310	*Arabidopsis*/*Mustard*	Chloroplast nucleoid^b^	Low PEP activity	Iron superoxide dismutase	SOD enzymes activity	Pfalz et al., 2006; Myouga et al., 2008; Steiner et al., 2011
PAP5/pTAC12^a^	AT2G34640	*Arabidopsis*/*Mustard*	Chloroplast and Nucleus^b^	Low PEP activity and	Structurally similar to the RAD23	Protein degradation in nuclear and unknown function in chloroplast	Pfalz et al., 2006; Chen et al., 2010; Steiner et al., 2011
PAP6/FLN1^a^	At3g54090	*Arabidopsis*/*Mustard*	Chloroplast nucleoid^b^	Low PEP activity	pfkB-type carbohydrate kinase	Unknown	Pfalz et al., 2006; Steiner et al., 2011; Gilkerson et al., 2012
PAP7/PTAC14^a^	AT4G20130	*Arabidopsis*/*Mustard*	Chloroplast^b^	Low PEP activity	SET domain	Unknown	Pfalz et al., 2006; Gao et al., 2011; Steiner et al., 2011
PAP8/pTAC6^a^	AT1G21600	*Arabidopsis*/*Mustard*	Chloroplast^b^	Low PEP activity	PHB_acc_N	Unknown	Pfalz et al., 2006; Steiner et al., 2011
PAP9/FSD1^a^	AT5G51100	*Arabidopsis*/*Mustard*	Chloroplast nucleoid^b^	Low PEP activity	Iron superoxide dismutase	SOD enzymes activity	Pfalz et al., 2006; Myouga et al., 2008; Steiner et al., 2011
PAP10/Trx Z^a^	AT3G06730	*Arabidopsis*/*Mustard*	Chloroplast stroma^b^	Low PEP activity	Thioredoxin	disulfide reductase activity *in vitro*	Pfalz et al., 2006; Arsova et al., 2010
PAP11/AtMurE^a^	AT1G63680	*Arabidopsis*/*Mustard*	Chloroplast^b^	Low PEP activity	Mur ligase family protein	Unknown	Pfalz et al., 2006; Garcia et al., 2008
pTAC7/PAP12^a^	AT5G24314	*Arabidopsis*/*Mustard*	Chloroplast^b^	Low PEP activity	Unknown	Unknown	Pfalz et al., 2006; Yu et al., 2013
FLN2^a^	AT1G69200	*Arabidopsis*/*Mustard*	Chloroplast nucleoid^b^	Low PEP activity	pfkB-type carbohydrate kinase	Unknown	Pfalz et al., 2006; Gilkerson et al., 2012; Huang et al., 2013
WHY1/pTAC1	AT1G14410	*Arabidopsis*/*Mustard*	Chloroplast and Nucleus^b^	Chloroplast genome rearrangement	DNA binding protein p24-related	Chloroplast genome rearrangement	Pfalz et al., 2006; Maréchal et al., 2009
WHY3/pTAC11	AT2G02740	*Arabidopsis*/*Mustard*	Chloroplast and Nucleus^b^	Chloroplast genome rearrangement	DNA binding protein p24-related	Maintaining chloroplast genome stability	Pfalz et al., 2006; Maréchal et al., 2009
pTAC4/Vipp1	AT1G65260	*Arabidopsis*/*Mustard*	Chloroplast^b^	Albino phenotype and Thylakoid biogenesis defects	PspA/IM30 family protein	Unknown	Pfalz et al., 2006
pTAC8	AT2G46820	*Arabidopsis*/*Mustard*	Chloroplast^b^	Thylakoid biogenesis	Curt1 domain	Thylakoid biogenesis	Pfalz et al., 2006; Armbruster et al., 2013
pTAC5	AT4G13670	*Arabidopsis*/*Mustard*	Chloroplast nucleoid^b^	Low PEP activity under heat stress	Peptidoglycan binding domain	Zinc-dependent disulfide isomerase activity	Pfalz et al., 2006; Zhong et al., 2013
pTAC17	AT1G80480	*Arabidopsis*/*Mustard*	Chloroplast	N.A.	CobW domain-containing	N.A.	Pfalz et al., 2006
pTAC18	AT2G32180	*Arabidopsis*/*Mustard*	Chloroplast	N.A.	Unknown domain	N.A	Pfalz et al., 2006
pTAC16	AT3G46780	*Arabidopsis*/*Mustard*	Chloroplast	No Phenotype	adh_short, Epimerase(1) NmrA(1) SPT2(1)	DNA binding protein	Pfalz et al., 2006
pTAC13	AT3G09210	*Arabidopsis*/*Mustard*	Chloroplast	N.A.	KOW and NusG domain	N.A.	Pfalz et al., 2006
PTAC9	AT4G20010	*Arabidopsis*/*Mustard*	Chloroplast	N.A	Single-stranded DNA binding	N.A	Pfalz et al., 2006
PTAC15	AT5G54180	*Arabidopsis*/*Mustard*	Chloroplast^b^	No Phenotype	Mitochondrial transcription termination factor	N.A.	Pfalz et al., 2006
GyrB	AT3G10270	*Arabidopsis*/*Mustard*	Chloroplast	Etiolated cytoledons	DNA_gyraseB,DNA_gyraseB_C, HATPase_c, Toprim	N.A	Pfalz et al., 2006; Pfalz and Pfannschmidt, 2013
GyrA	AT3G10690	*Arabidopsis*/*Mustard*	Chloroplast	Embryo lethal	DNA_gyraseA_C; DNA_topoisoIV	N.A.	Pfalz et al., 2006; Pfalz and Pfannschmidt, 2013
PolA	AT3G20540	*Arabidopsis*/*Mustard*	Chloroplast	N.A.	3_5_exonuc, DNA_pol_A	N.A.	Pfalz et al., 2006
rpL12	AT3G27830	*Arabidopsis*/*Mustard*	Chloroplast	N.A.	Ribosomal_L12		Pfalz et al., 2006
RABE1B	AT4G20360	*Arabidopsis*/*Mustard*	Chloroplast	N.A	ATP_bind; CbiA; cobW; GTP_EFTU; GTP_EFTU_D2; GTP_EFTU_D3; Miro;	N.A.	Pfalz et al., 2006
rpL29	AT5G65220	*Arabidopsis*/*Mustard*	Chloroplast	N.A	Ribosomal protein L29	N.A.	Pfalz et al., 2006
PTK/cpCK2	AT2G23070	*Mustard*	Chloroplast^b^	N.A	CK2 kinase domain	Protein kinase activity	Loschelder et al., 2004
CSP41b	AT1G09340	*Mustard*	Chloroplast	Pale green seedlings	RNA Binding	Chloroplast ribosomal RNA metabolism	Pfannschmidt et al., 2000; Ogrzewalla et al., 2002; Loschelder et al., 2004
CSP41A	AT3G63140	*Mustard*	Chloroplast	No Phenotype	RNA Binding	Chloroplast ribosomal RNA metabolism	Pfannschmidt et al., 2000; Loschelder et al., 2004
Atann4	AT2G38750	*Mustard*	Chloroplast	N.A.	Calcium ion binding,calcium-dependent phospholipid binding	N.A.	Loschelder et al., 2004
ETCHED1	AT1G68730	*Maize*	Chloroplast^b^	N.A.	Zim17-type zinc finger protein	N.A.	da Costa e Silva et al., 2004
AT2G35605	AT2G35605	*Spinach*	Chloroplast^b^	N.A.	SWIB/MDM2 domain	N.A.	Melonek et al., 2012
AT2g02060	AT2g02060	*Spinach*	Chloroplast	N.A.	N-CoR and TFIIIB DNA binding domains	N.A.	Melonek et al., 2012
AT3G14320	AT3G14320	*Spinach*	Chloroplast	N.A.	AP2 DNA binding	N.A.	Melonek et al., 2012
AT2G27430	AT2G27430	*Spinach*	Chloroplast	N.A.	Armadillo-type fold, armadillo-like helical	N.A.	Melonek et al., 2012
AT5G02320	AT5G02320	*Spinach*	Chloroplast	N.A.	N-CoR and TFIIIB DNA binding domains	N.A.	Melonek et al., 2012
AT5G36780	AT5G36780	*Spinach*	Chloroplast	N.A.	RecF/RecN/SMC N-terminal domain	N.A.	Melonek et al., 2012
MFP1	AT3G16000	*Spinach*	Chloroplast^b^	N.A.	DNA binding	N.A.	Melonek et al., 2012

* *Species that chloroplast proteomics experiments were performed are indicated*.

** *Localization information is from GFP- fusion data and or chloroplast proteomics data /immune analysis*.

*** *Protein domain information is from PPDB database*.

**** *Molecular function data is given based on the reference. N.A. means that its detailed molecular function remains unclear*.

***** *The phenotypes of the knockout lines in Arabidopsis are indicated. N.A. means that the phenotype remains unclear*.

a *These factors are essential for PEP activity*.

b *Localization information is from individual GFP-fusion experiment or immune analysis*.

**Figure 1:**
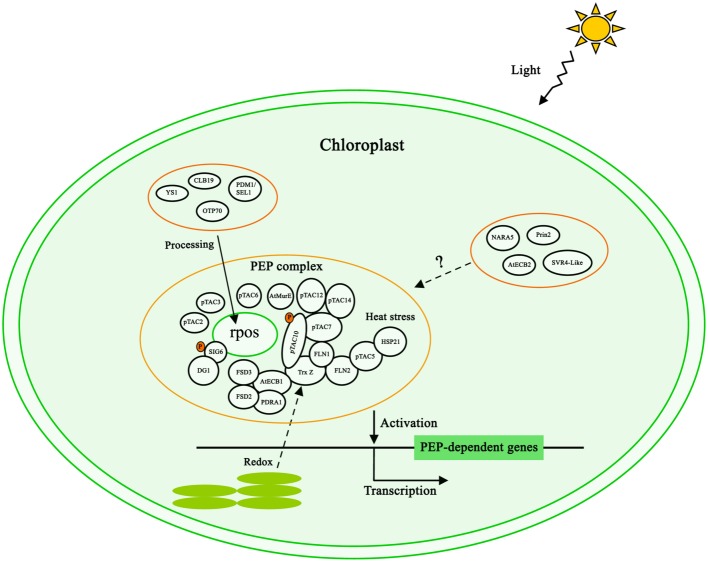
**Model of the PEP complex regulating chloroplast transcription**. The reported interactions of PEP components are included. Light, redox status, phosphorylation and heat stress are involved in chloroplast transcription. Several PPR proteins including CLB19, PDM1/SEL1, OTP70, and YS1 are involved in the processing of PEP core subunit transcripts to regulate PEP activity. AtECB2, Prin2, SVR4-Like and NARA5 are also important for plastid gene expression, but their mechanisms are unclear.

Based on proteomic analysis and protein interaction investigation, the TAC complex contains at least 50 proteins of which 46 are nuclear-encoded (Tables 1, 2). These nuclear-encoded proteins can be classified into several groups including DNA/RNA binding proteins, thioredoxin proteins, kinases, ribosome proteins and proteins with unknown function (Table 1). Yeast two-hybrid and other biochemical assays revealed the relationship of some proteins in the PEP complex (Figure 1). The interactions between these PAPs are consistent with the biochemical experiments that identified these proteins in the PEP complex under the stringent condition (Steiner et al., 2011). Currently, proteins directly interacting with the PEP core subunits have not been identified in the PEP complex. Immunoprecipitation analysis demonstrated that pTAC3 is associated with the *rpo* subunits (Yagi et al., 2012). However, the direct interaction between pTAC3 and PEP core subunits has not been verified.

**Table 2:** **Individual mutant analysis identifies several factors which affect PEP-dependent chloroplast transcription in higher plant**.

**Gene name**	**AGI number**	Phenotype^*^	Localization^**^	Molecular phenotype^*^	Protein domain^***^	Molecular function^****^	**References**
DG1^a^	AT5G67570	Delayed green	Chloroplast	Low PEP activity	PPR domain	Interacts with sig6	Chi et al., 2008, 2010
AtECB1/MRL7/SVR4^a^	AT4G28590	Albino	Chloroplast nucleoid	Low PEP activity	Thioredoxin-Like fold	Thioredoxin activity	Qiao et al., 2011; Yu et al., 2014;
PDRA1^a^	AT5G48470	Yellowish	Chloroplast nucleoid	Low PEP activity	U.K.	Function unknown	Qiao et al., 2013
NARA5^b^	AT4G27600	Yellowish	Chloroplast	Low PEP activity	pfkB-type carbohydrate kinase	U.K.	Ogawa et al., 2009
MRL7-Like/SVR4-Like/AtECB1-Like^b^	AT2G31840	Albino	Chloroplast stroma	Low PEP activity	Thioredoxin-like fold	U.K.	Qiao et al., 2011; Powikrowska et al., 2014
Prin2^b^	AT1G10522	Yellowish	Chloroplast nucleoid	Low PEP activity	U.K.	U.K.	Kindgren et al., 2012
AtECB2/VAC1^b^	AT1G15510	Albino	Chloroplast	Low PEP activity, accD and Ndhf editing defect	PPR domain	RNA editing	Yu et al., 2009; Tseng et al., 2010
OTP70^c^	AT4G25270	Pale green	Chloroplast	Low PEP activity, splicing of the plastid transcript rpoC1	PPR domain	RNA splicing	Chateigner-Boutin et al., 2011
YS1^c^	AT3G22690	Delayed green	Chloroplast	Low PEP activity, rpoB editing defect	PPR domain	RNA editing	Zhou et al., 2009
SEL1/PDM1^c^	AT4G18520	Albino	Chloroplast	Low PEP activity, rpoA processing defect	PPR domain	RNA processing	Pyo et al., 2013
CLB19^c^	AT1G05750	Albino	Chloroplast	Low PEP activity, RNA editing defect	PPR domain	RNA editing	Chateigner-Boutin et al., 2008

* *Observed phenotype and Molecular phenotype of inactivation mutants is given*.

** *Localization information is from GFP- fusion data*.

*** *Protein domain information is from PPDB database*.

**** *Molecular function data is given based on the reference. U.K. means that its detailed molecular function remains unclear*.

a *These factors are associated with the PAPs or the core subunits*.

b *These factors regulate plastid transcription with unknown mechanism*.

c *These factors indirectly affect PEP activity through regulating the processing of chloroplast transcripts encoding the core subunits*.

### Proteins in the PEP complex with DNA/RNA binding domain

The eukaryotic transcriptional machinery consists of RNA polymerases and various DNA binding proteins, such as transcription factors. These DNA-binding proteins recognize the promoter to regulate downstream gene transcription. In the TAC complex, there are at least 14 proteins with DNA-binding domains (Table 1) (Pfalz et al., 2006; Steiner et al., 2011; Pfalz and Pfannschmidt, 2013). pTAC3 belongs to the SAP protein family. The *ptac3* mutant exhibits an albino phenotype with reduced PEP-dependent plastid transcription. It is unclear yet if pTAC3 can bind to a specific DNA region in order to regulate plastid gene transcription (Yagi et al., 2012). pTAC6 is essential for chloroplast transcription (Pfalz et al., 2006) since the expression of the *psbA* gene was barely detectable in the *ptac6* mutant, compared with that in *ptac2* and *ptac12* (Pfalz et al., 2006). It is likely that pTAC6 is a specific regulator for *psbA* (Pfalz et al., 2006), however, to date its function remains enigmatic. In bacteria, there exist two transcription termination mechanisms; Rho-independent transcription termination and Rho-dependent termination. The mitochondrial transcription termination factor (mTERF) family was identified to regulate mitochondrial gene expression including transcription termination (Kleine, 2012). pTAC15 is a member of the mTERF protein family (Pfalz et al., 2006). Whether it can terminate the transcription of PEP-dependent plastid genes needs to be verified.

The TAC complex contains at least six RNA-binding proteins including ZmWhy1, pTAC10, the elongation factor EF-Tu, and three ribosomal proteins, S3, L12-A, and L26 (Table 1). Whirly proteins belong to a small nuclear transcription factor family commonly found in plants. In *Arabidopsis*, pTAC1/AtWhy1 and pTAC11/AtWhy3 can bind DNA (Xiong et al., 2009). They are required to maintain the stability of the plastid genome (Maréchal et al., 2009). The whirly 1 ortholog in *maize* (ZmWHY1/pTAC1) can bind both RNA and DNA, and co-immuno-precipitated with chloroplast RNA splicing 1 (CRS1) (Prikryl et al., 2008). pTAC10 contains a S1 domain and has RNA binding activity in tobacco (Jeon et al., 2012), and it may be one substrate of chloroplast-target casein kinase 2 (cpCK2) (Reiland et al., 2009). The phosphorylation of pTAC10 may affect its RNA binding. The detailed function of the elongation factor EF-Tu and the ribosomal proteins S3, L12-A, and L26 in chloroplast is not reported. The existence of these RNA-binding proteins, however, suggests that there exists a translation subdomainin the TAC/nucleoid.

### Connections of regulatory modules with the RNA polymerase

Light plays highly important roles in the regulation of plastid gene transcription. The majority of PAPs (Pfalz and Pfannschmidt, 2013) and most sigma factor genes of higher plants are light-induced (Lerbs-Mache, 2011). Plastome-wide PEP-DNA association is also a light-dependent process (Finster et al., 2013). In plants, light plays an important role in almost every facet of plant growth and development through the action of photoreceptors. Interestingly, pTAC12 is an intrinsic subunit of the PEP complex (Pfalz et al., 2006; Steiner et al., 2011), but it was also identified as HEMERA and localized in both the nucleus and the chloroplast (Chen et al., 2010). pTAC12/HEMERA was considered as a proteolysis-related protein involved in phytochrome signaling in the nucleus (Chen et al., 2010). Its function in the PEP complex is unknown so far, but it was uncovered that pTAC12 interacts with pTAC14 in the yeast-two-hybrid system (Gao et al., 2011) suggesting that these two proteins might be also interaction partners in the native complex.

Chloroplasts are the site of photosynthesis that also produces reactive oxygen species (ROS). During photosynthesis, unbalanced excitation of the two photosystems affects the redox state of the electron transport chain which in turn serve as signals for plant acclimation responses. The PEP complex is a major target of such photosynthetic redox signals (Dietz and Pfannschmidt, 2011). Thioredoxin z (Trx Z) is a novel thioredoxin protein with disulfide reductase activity *in vitro*. It interacts with two fructokinase-like proteins FLN1 and FLN2 in the yeast two hybrid system and is also a component of the PEP complex (Pfalz et al., 2006; Steiner et al., 2011) (Figure 1). Trx-Z mediated redox change of FLN2 during light–dark transitions (Arsova et al., 2010). Recent studies identified AtECB1/MRL7 as a thioredoxin-fold like protein with thioredoxin activity (Yu et al., 2014) that interacts with Trx Z in the PEP complex (Powikrowska et al., 2014; Yu et al., 2014). These two proteins thus may form a functional module to mediate redox signaling from thylakoids toward the RNA polymerase but the functional details of these interactions are completely unknown. Further redox mediators might be Fe Superoxide Dismutase 2 (FSD2) and FSD3, two iron superoxide dismutases, and PRDA1 is a chloroplast protein without any known domain. *prda1* and *fsd2 fsd3* knock out mutants are highly sensitive to oxidative stress (Myouga et al., 2008; Qiao et al., 2013). These proteins, therefore, may act as ROS scavengers in order to protect the PEP complex. The interactions between AtECB1 and PRDA1, FSD2, FSD3 suggest that the redox signaling pathway and ROS scavengers are eventually associated.

Protein phosphorylation is a very important post-translational modification in eukaryotic cells that regulates many cellular processes. In chloroplast, the phosphorylation of chloroplast proteins affects photosynthesis, metabolic functions and chloroplast transcription (Baginsky and Gruissem, 2009). The PEP complex appears to interact with a so-called plastid transcription kinase (PTK), named cpCK2 (Ogrzewalla et al., 2002). The *Arabidopsis* sigma factor 6 was reported to be phosphorylated by cpCK2 (Schweer et al., 2010a). Furthermore, pTAC5, pTAC10, and pTAC16, were also predicated to be phosphorylated by cpCK2 (Reiland et al., 2009). The enzyme activity of cpCK2 was inhibited by GSH, which suggests that cpCK2 is generally under SH-group redox regulation (Baginsky et al., 1999; Turkeri et al., 2012). Biochemical analyses of *mustard* seedlings during photosynthetic acclimation suggested that redox signals in chloroplasts are linked to chloroplast transcription *via* the combined action of phosphorylation and thiol-mediated regulation events (Steiner et al., 2009). Proteins related with phosphorylation and redox signaling are closely located in the PEP complex which is in agreement with the results of the physiological studies for plastid gene expression.

Heat stress is a major abiotic factor for plants, that leads to severe retardation in plant growth and development. To maintain the process of chloroplast transcription under heat stress and to support the survival of the plant, the chloroplast transcriptional machinery needs to deal with heat stress to a certain extent. The protein pTAC5 is a C4-type zinc finger DnaJ protein with disulfide isomerase activity. Its expression is induced by heat stress (Zhong et al., 2013) and, subsequently, pTAC5 and Heat Shock Protein 21 (HSP21) form a heterocomplex, although they are not PAP members of the PEP complex (Zhong et al., 2013). pTAC5 as well as HSP21 may protect chloroplast transcription under heat stress.

### Other nuclear encoded factors that regulate PEP activity

In addition to the intrinsic components of PEP complex, multiple additional factors were identified to regulate the processing of PEP core subunit transcripts and PEP activity by individual mutant analysis. Both *Chloroplast Biogenesis19* (*CLB19*) (Chateigner-Boutin et al., 2008) and *Pigment-Deficient Mutant 1*(*PDM1*) (Wu and Zhang, 2010; Yin et al., 2012) genes encode pentatricopeptide repeat proteins. CLB19 is involved in the editing of the *rpoA* transcript (Chateigner-Boutin et al., 2008), while *PDM1* is associated with *rpoA* polycistronic for *rpoA* cleavage (Wu and Zhang, 2010; Yin et al., 2012). Recent investigations demonstrated that PDM1/Seedling Lethal1 (SEL1) was also involved in *accD* RNA editing (Pyo et al., 2013). The PPR protein OTP70 was reported to affect the splicing of the *rpoC1* transcript (Chateigner-Boutin et al., 2011). The gene *Yellow Seedling 1* (*YS1*) encoding a PPR-DYW protein is required for editing of *rpoB* transcripts (Zhou et al., 2009). The common feature of the *Arabidopsis* knockout lines for all these proteins is that the plastid expression pattern in these mutants is similar to that of *rpo*-gene knock-out mutants in tobacco (Chateigner-Boutin et al., 2008, 2011; Zhou et al., 2009; Wu and Zhang, 2010; Pyo et al., 2013).

Functional analyses revealed that several proteins including *Arabidopsis* Early Chloroplast Biogenesis 2 (AtECB2) (Yu et al., 2009), Plastid redox insensitive 2 (Prin2) (Kindgren et al., 2012), SVR4-Like (Powikrowska et al., 2014), and NARA5 (Ogawa et al., 2009), are also essential for PEP-dependent chloroplast transcription. However, it is unclear if they are directly associated with the PEP complex. AtECB2 encodes a pentatricopeptide repeat protein, and is involved in editing of *accD* and *ndhF* chloroplast transcripts (Yu et al., 2009; Tseng et al., 2010). The defective editing in *ecb2* is unlikely to affect PEP-dependent plastid gene expression. How AtECB2 affects plastid gene expression is still unclear. NARA5 encodes a chloroplast-localized phosphofructokinase B-type carbohydrate kinase family protein, which might be involved in massive expressions of plastid-encoded photosynthetic genes in *Arabidopsis* (Ogawa et al., 2009). The Prin2 is a small protein possibly involved in redox-mediated retrograde signaling in chloroplast (Kindgren et al., 2012) and the SVR4-like is a homolog of AtECB1/SVR4/MRL7, encoding a chloroplast protein essential for proper function of the chloroplast in *Arabidopsis* (Powikrowska et al., 2014). All these proteins may reversibly associate with the PEP complex but detailed studies are necessary to understand their functional roles and connections with the RNA polymerase. Alternatively, these proteins may act as signaling factors in order to mediate environmental stimuli and plastid gene expression.

## Concluding remarks

Plants grow under very different environment conditions and photosynthesis is the major function of chloroplast which is important for plant growth and development. Plastid gene expression is essential for chloroplast development and normal functions including photosynthesis. The PEP complex is the major RNA polymerase activity in mature chloroplasts. Proteomic and genetic analyses identified that at least 50 nuclear-encoded proteins in higher plant are important for PEP dependent plastid gene expression. These proteins may form several functional modules within the nucleoid or TAC in order to mediate plastid gene expression in response to light, redox changes, phosphorylation and heat stress or to protect the PEP complex from ROS damage. The large number of nuclear-encoded proteins reveals the complexity of plastid gene expression and regulation that is greatly different from the gene expression in the nucleus or in prokaryotes. However, the current knowledge about plastid transcription is quite limited and the investigation of the relationship between transcription, post-transcriptional processing as well as translation in the nucleoid could provide novel insights into chloroplast gene expression.

### Conflict of interest statement

The authors declare that the research was conducted in the absence of any commercial or financial relationships that could be construed as a potential conflict of interest.
